# Airborne Detection of H5N8 Highly Pathogenic Avian Influenza Virus Genome in Poultry Farms, France

**DOI:** 10.3389/fvets.2018.00015

**Published:** 2018-02-13

**Authors:** Axelle Scoizec, Eric Niqueux, Rodolphe Thomas, Patrick Daniel, Audrey Schmitz, Sophie Le Bouquin

**Affiliations:** ^1^Avian and Rabbit Epidemiology and Welfare Unit, ANSES, French Agency for Food Environmental and Occupational Health Safety, Ploufragan, France; ^2^Avian and Rabbit Virology, Immunology and Parasitology Unit, ANSES, French Agency for Food Environmental and Occupational Health Safety, Ploufragan, France; ^3^Laboratoire des Pyrénées et des Landes, Mont de Marsan, France

**Keywords:** avian influenza, highly pathogenic avian influenza, H5N8, clade 2.3.4.4, airborne, transmission, ducks, chickens

## Abstract

In southwestern France, during the winter of 2016–2017, the rapid spread of highly pathogenic avian influenza H5N8 outbreaks despite the implementation of routine control measures, raised the question about the potential role of airborne transmission in viral spread. As a first step to investigate the plausibility of that transmission, air samples were collected inside, outside and downwind from infected duck and chicken facilities. H5 avian influenza virus RNA was detected in all samples collected inside poultry houses, at external exhaust fans and at 5 m distance from poultry houses. For three of the five flocks studied, in the sample collected at 50–110 m distance, viral genomic RNA was detected. The measured viral air concentrations ranged between 4.3 and 6.4 log_10_ RNA copies per m^3^, and their geometric mean decreased from external exhaust fans to the downwind measurement point. These findings are in accordance with the possibility of airborne transmission and question the procedures for outbreak depopulation.

## Introduction

A H5N8 clade 2.3.4.4 strain of highly pathogenic avian influenza (HPAI) virus (HPAIV) was first detected in France in November 2016. Until the 3rd of March 2017, 348 cases of HPAI H5N8 and 136 cases of HPAI H5Nx strain closely related to HPAIV H5N8 were detected in poultry, with 80% of cases occurring in waterfowl farms (mainly duck farms) (1). In the area affected by the outbreak (zones from 0 to 5 km distance from a poultry case), the mean proportion of poultry farms affected was around 15 and 24% where the poultry farm density was greater than 1/km^2^. In the southwestern region of France, the virus spread rapidly especially in high poultry farm density zones, despite the implementation of routine control measure. This rapid regional spread and the proportion of farms affected in some areas, drove us to question the potential role of airborne transmission in HPAI H5N8 viral spread.

The capacity of poultry to transmit influenza virus *via* the airborne route, was evidenced by experimental studies in chickens infected with the H5N1 HPAIV strain (2, 3) and was further supported by field studies as the ones detailed below. Thus, the detection and isolation of strains of AIV in air samples, with particles sizes partly compatible with respiratory contamination, in wet poultry markets could explain human infections reported after a visit of a wet poultry market without any direct contact with live poultry or poultry stalls (4–6). Detection of different AIV strains, with or without quantification, have been performed on air samples collected outside, inside and downwind from infected poultry premises, up to 59 m for low pathogenic strains and up to 1,000 m for highly pathogenic ones (7–9) and occurred partly on particles respirable fraction. Isolation of HPAIV H5N2 clade 2.3.4.4 has been performed on air samples collected inside, 5 m outside and even 70–150 m outside from poultry barns (8, 9).

The capacity of poultry flock to be infected through the airborne route is strongly suggested by epidemiological studies. For example, pig farm proximity to turkey premises has been associated with turkey seropositivity to swine-origin influenza A virus (IAV) and the detection and quantification of swine IAV in air samples collected inside and outside swine barns (10), support the hypothesis of airborne transmission (11). Modeling studies on the outbreak of HPAI H7N7 in the Netherlands in 2003, estimated the contribution of a possible wind-mediated mechanism to the total amount of spread to be around 18% (12) and showed that the wind-borne route could contribute substantially to the spread over short distance ranges, explaining, for example, 24% of the transmission over a distance up to 25 km (13).

The first observations of the French H5 clade 2.3.4.4 epizootic short distance diffusion (<10 km) (14) are compatible with a contribution of wind-born transmission to the spread when compared with the Dutch H7N7 2003 outbreak. Thus the objective of this study was to determine whether AIV could be detected in air samples collected inside, outside, and downwind from poultry barns infected by H5N8 HPAIV under field conditions. This study was designed and performed as part of a rapid outbreak response.

## Materials and Methods

### Flock Selection/Description

**Figure 1:**
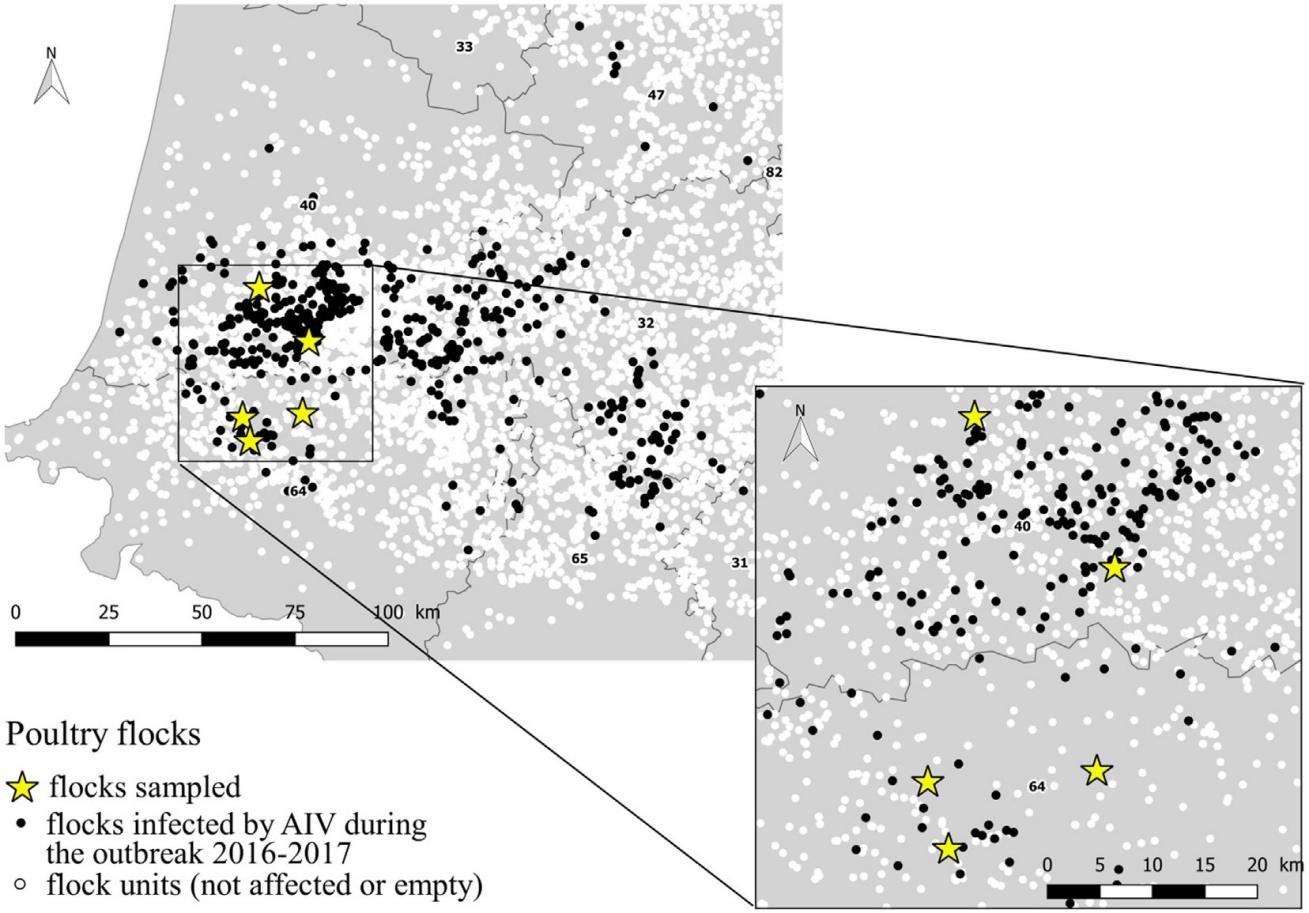
Location of the sampled flocks within the area affected by the AIV 2016–2017 outbreak.

The study was conducted in January and March 2017. The selection of flocks was carried out in collaboration with departmental animal health authorities regarding the confirmed infected status, the not-yet depopulation of flocks and the agreement of the farmer, at the time of the field team availability. Three duck flocks (A, B, and C) and two chicken flocks (D and E), located in Landes and Pyrénées Atlantiques departments were selected. All selected flocks had an officially confirmed diagnosis of HPAI H5N8 at the time of sampling, according to the European diagnostic manual for avian influenza (15). Sampling was performed 2–7 days after confirmation date. At the sampling event, three of the five selected flocks were confined totally in-house (C, D, and E). Loading for culling occurred during the sampling process for one flock (E). A part of the ducks for the flocks A and B, had still an access to the open free range at the sampling event. Characteristics of flocks are summarized in Table 1 and their location within the affected region presented in the Figure 1.

**Table 1:** Attributes of flocks studied and environmental conditions at sampling events.

Farm ID	French depart.^a^	Specie/type	House	Flock initial size	House poultry density^b^	Positive confirmation date^c^ (dd/mm/yyyy)	Proportion of positive pools^d^	Clinical signs	Air sampling date (dd/mm/yyyy)	Sampling location/distance (m)	Ambient temperature (°C)	Wind velocity (km/h)
A	40	Ducks/PAG^e^	Tunnel^f^	2,500	7/m^2^	29/01/2017	2/2	Mortality/symptoms	31/01/2017	Inside	NR	<5
											NR	
										External exhaust fans	NR	
											20	
										Outside 5 m		
										Downwind 50 m		

B	64	Ducks/PAG	Tunnel	3,000	0.5/m^2^	09/03/2017	5/24	None	16/03/2017	Inside	NR	10
											NR	
										External exhaust fans	NR	
										Outside 5 m	24	
										Downwind 80 m		

4	64	Ducks/FF^G^	Barn	800	2.5/m^2^	11/03/2017	12/12	None	16/03/2017	Inside	NR	<5
											NR	
										External exhaust fans	NR	
										Outside 5 m	17	
										Downwind 60 m		

D	40	Chickens/grow	Barn	4,000	1/m^2^	14/03/2017	2/2	Mortality/symptoms	21/03/2017	Inside	NR	<5
											NR	
										External exhaust fans	NR	
											12	
										Outside 5 m		
										Downwind 50 m		

E	64	Chickens/grow	Barn	4,400	8/m^2^	18/03/2017	8/8	Mortality/symptoms	22/03/2017	Inside	NR	≈0
										Loading for culling	NR	
											2	
										Downwind 110 m		

*^a^Department is an administrative division unit in France (the median land area of French metropolitan departments is 5,960 km^2^)*.

*^b^At sampling event*.

*^c^Date of the official sampling that permitted to confirm the avian influenza H5 infection of the flock*.

*^d^Proportion of pools of five swabs (cloacal or oropharyngeal) positive to rRT-PCR targeting the matrix gene at the official sampling*.

*^e^PAG, growing ducks for “foie gras” production*.

*^f^Tunnel: open sided tunnel*.

*^g^FF (“foie gras” production)*.

*NR, not recorded; FF, force feeding period; PAG, prêts à gaver*.

### Air Sampling Procedures and Sampling Scheme

To detect AIV genome in aerosols, air samples were collected using a cyclone-based bioareosol sampler, Coriolis^®^μ microbial air sampler (Bertin Technologies, St-Quentin en Yvelines, France): 300 L/min, 10 min/sample, in 10–12 mL of 0.005% Triton X-100 (Sigma Aldrich) solution prepared in demineralized water and placed into a sterile sampling cone. The collected sample was poured directly after collection from the sampling cone into a sterile 50 mL tube.

After each sample collection, the air sampler was cleaned and disinfected, the cone removed and the sample stored at 0–4°C. The disinfection was performed by spraying Aniospray Surf 29 (Laboratoires Anios, France) on external surface and inside and outside the air intake and the aspiration tube. The samples were transported to a nearby laboratory (from accredited laboratories national network) within 12 h where they were stored at −80°C until testing.

For each flock, air samples were collected in the following order: downwind from the barn at 50–110 m distance, at 5 m distance, at external exhaust fans and finally inside the barn. For one flock (E), the loading of the flock for culling started during the sampling process, the air samples were collected downwind at 110 m distance, inside the barn and at 1 m distance from the animal transport truck. One control sample was collected at 5 km distance from any poultry farm. The sampler was placed directly against the exhaust fans and on the ground for the other sampling locations.

### Detection and Quantification of AIV RNA Genome

Collected air samples were concentrated using a Amicon^®^ Ultra-15 30K centrifugal filter device (Merck Millipore Ltd., Ireland). After centrifugation (for 30 min at 5,000 *g*), RNA was purified from 200 µL eluate using the RNeasy Mini Kit© (Qiagen GmbH, Hiden, Germany), and 2 µL RNA extract from the 50 µL obtained from purification was tested by real-time reverse-transcription polymerase chain reaction (rRT-PCR) targeting the matrix gene (M gene) of avian influenza type A viruses, as previously described by Ref. (16, 17). Samples with a detection of M gene signal were tested by subtype specific H5 rRT-PCR (16, 18). We will refer to samples with a detection of viral genome signal by rRT-PCR as positive in the text that follows. For the positive samples, the number of M gene copies in the volume of analyzed sample is estimated from the cycle threshold (Ct) value obtained in RT-PCR, according to a calibration curve relating decimal dilution series of a synthetic RNA transcript of known concentration (determined by fluorimetric quantitation) to Ct values: each dilution point of the RNA transcript was tested twice.

For each sample, the number of AIV M gene copies per m^3^ air was calculated according to the formula:

$$\text{M gene copies}/{\text{m}}^{\text{3}}=\text{M gene copies PCR }\times \;(\text{Vextract}÷\text{Vpcr})\;÷(U\times t),$$

where Vextract is the sample final reduced volume obtained after centrifugation and RNA extraction, Vpcr is the volume analyzed by RT-PCR, *U* is the air flow rate (m^3^ per min), and *t* is the sampling duration (min).

### Ethic Statement

Air sampling was performed with the permission of the farmers and the departmental animal health authorities.

## Results

### Detection of HPAI Viral Genome in Air Samples

In the control sample, no viral genome signal was detected by M gene rRT-PCR. All positive air samples detected in this study, were both positive by M gene and H5 subtype rRT-PCR. All air samples collected inside (5/5), at external exhaust fans (4/4), 5 m outside the barn (4/4) were positive. Three of the five samples collected downwind from the barn were also positive (Table 2). Regarding samples collected downwind, the positive samples correspond to the flocks with clinical signs (mortality) and to an ambient temperature at sampling event of 2, 12, and 20°C and the negative samples to the asymptomatic flocks and to an ambient temperature of 17 and 24°C. The two flocks (B and C) with no detection of viral genome in air sample collected downwind also had low housing poultry densities. The sample collected during the animal loading was positive. In the five flocks studied, all air samples collected inside and at least one sample collected outside at 5–110 m distance from the barn were positive.

**Table 2:** Detection of influenza virus genome in air samples by rRT-PCR inside and outside poultry barns infected by HPAI subtype H5N8 clade 2.3.4.4.

Farm ID	Specie/type	M gene rRT-PCR Ct value/H5 gene rRT-PCR Ct value				
		Inside	External exhaust fans	Outside 5 m	Downwind (distance in m)	Loading for culling
A	Ducks/PAG^a^	32.4/34.9	32.7/35.8	32.3/36.1	33.6/35.4 (50)	NT
B	Ducks/PAG	35.6/39.7	31.2/34.8	33.9/35.8	Not detected (80)	NT
C	Ducks/FF^b^	29.8/30	31/30.7	30.5/31.1	Not detected (60)	NT
D	Chickens/grow	34.9/35.3	33.1/34.4	33.1/36.2	34.2/38.8 (50)	NT
E	Chickens/grow	31.5/32	NT	NT	34.2/37.5 (110)	28.7/29.3

*^a^PAG, growing ducks for “foie gras” production*.

*^b^FF (“foie gras” production)*.

*Ct, cycle threshold; PAG, prêts à gaver; FF, force feeding period; NT, not tested*.

### Quantification of HPAI Viral Genome in Air Samples

**Figure 2:**
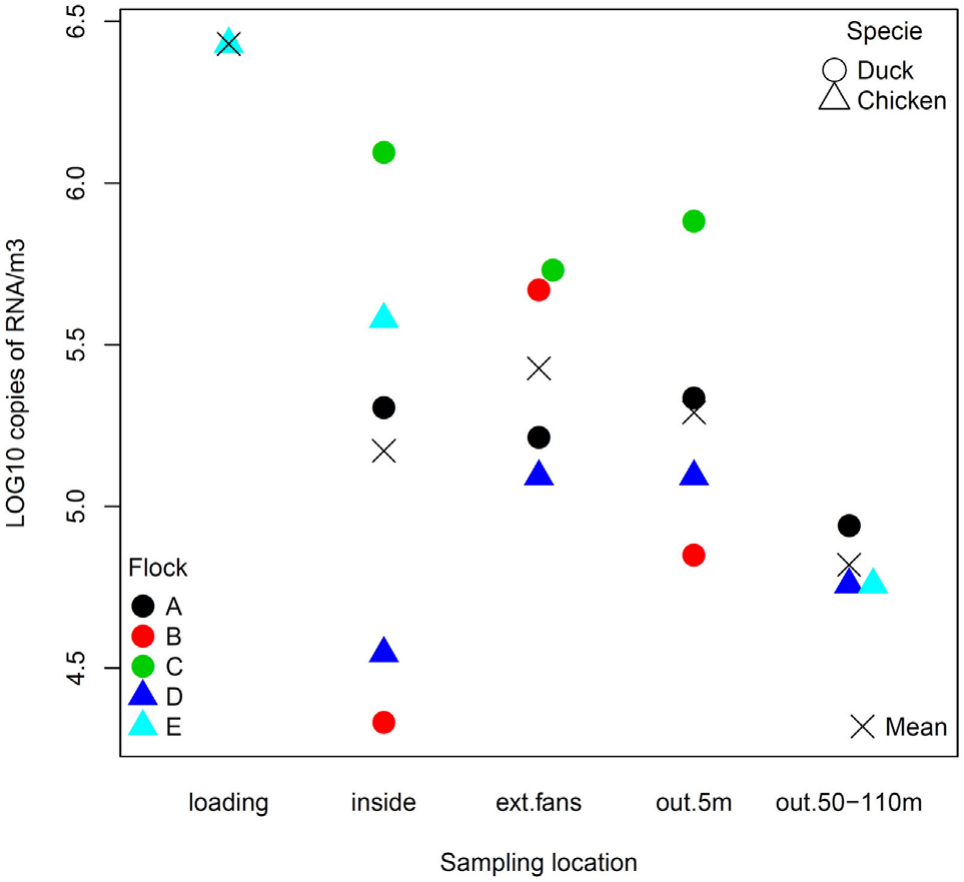
Viral RNA concentration (log_10_ copies of RNA per m^3^) of positive air samples by sampling location.

The quantity of virus (expressed in log_10_ RNA copies per m^3^) estimated in positive air samples, ranged from 4.33 to 6.09 and from 4.54 to 6.43, in duck and chicken flocks, respectively (Figure 2; Table 3). The maximum air viral RNA concentration was found at the animal loading point. For two of four flocks (one duck, one chicken, flocks B and D), the concentration found at the external fans was higher than inside the barn. There was a higher concentration variability between flocks (Figure 2; Table 3) for the samples collected inside barns than at the other sampling locations. The two lowest air concentrations measured inside barns corresponded to the lowest house poultry densities flocks (B and D) at sampling event. Furthermore the lowest of these two air concentrations mentioned above also corresponded to the flock (B) with the lowest proportion of pools of five swabs positive by rRT-PCR targeting the matrix gene (Table 1). The highest concentrations measured inside and at the short distance outside (external exhaust fans and 5 m distance) corresponded to the flock (C) of ducks at the force feeding period. Outside of the barns, there was a decrease of the geometric mean of positive air sample RNA concentrations measured against an increasing distance from the barns (Table 3).

**Table 3:** Quantity (RNA copies/m^3^ of air) of H5N8 HPAIV in positive sampling events collected inside and outside duck and chicken premises.

Sampling location	Duck				Chicken			
	*n*	GM	GSD	max	*n*	GM	GSD	max
Inside	3	1.76E + 05	7.6	1.25E + 06	2	1.15E + 05	5.4	3.79E + 05
External exhaust fans	3	3.46E + 05	1.9	5.38E + 05	1	1.24E + 05	–	–
Outside 5 m	3	2.27E + 05	3.3	7.64E + 05	1	1.24E + 05	–	–
Downwind (50–110 m)	1	8.72E + 04	–	–	2	5.73E + 04	0	–
Loading for culling					1	2.69E + 06	–	–

*GM, geometric mean; GSD, geometric standard deviation*.

## Discussion

The rapid spread of H5N8 or H5Nx HPAI clade 2.3.4.4 virus during the winter of 2016–2017 in South West France raised questions about the possibility of airborne transmission contribution to the global spread. As a first step in the investigation of airborne transmission hypothesis, we detected H5 gene viral RNA from air samples collected inside, outside and downwind of H5N8 HPAI infected poultry facilities and this detection occurred inside and outside poultry facilities in all of the five flocks studied.

The percentage of actively infected birds, the poultry density and environmental conditions inside and outside barns at the time of sampling, were expected to influence the detection and the concentration of viral genome in air samples. This seems to be particularly the case for the measurement inside the barns. The decrease of positive air sample proportion, as well as the decrease of viral genome concentration in air samples between the samples collected inside or at short distance outside poultry facilities and the ones collected at 50–110 m distance, likely reflect decreasing virus concentration by dilution as a function of distance from the source. The time of sampling, which took place late morning (10 a.m.) for the flocks D and E and early afternoon (2 p.m.) for the other flocks (A, B, and C), could have also influenced the results due to the ambient temperature. Indeed, it could have contributed to the no detection of viral genome at 50–110 m distance for two of the three flocks collected early afternoon.

The levels of viral detection (proportion of positive samples and positive air sample viral RNA concentrations) were comparable to the ones found around H5N2 clade 2.3.4.4 HPAI poultry facilities during the 2015 spring outbreaks in the United States (8) and higher than the results around LPAI poultry facilities in the Netherlands (7) and at live poultry markets in China (4, 6). The virus viability in the air samples collected could not be investigated in this study, due to the sample processing (nature of the solution used). However, based on previous studies with different strains of AIV (6) or the same clade of AIV (8), we hypothesize that viable virus was likely captured in our sampling given the high levels of viral RNA concentrations.

For the airborne transmission of HPAIV to potentially occur, it would require not only the transport of viable virus on aerosolized particles, but also the capacity of viral contaminated particles to infect birds. The fact that experimentally, H5N1 HPAI airborne transmission has been performed with chickens (2, 3, 19) with air viral genome concentrations (all air fractions included) comparable to Ref. (3) our findings, is in favor of the hypothesis of infective capacity of the contaminated aerosolized particles present in the positive air samples collected. Even considering that the infectivity of AIV, considering the infectious dose, is both host-dependent and virus strain-dependent (20–23), the fact that a low mean bird infectious dose (<2–3 log_10_ EID_50_) by intranasal route has been determined with H5 HPAIV clade 2.3.4.4 (H5N8 and H5N2) United States index viruses in Pekin ducks and Chinese geese (24) and that the infectivity of AIV can be much higher (30 times) by aerosol route as compared with intranasal route, as established for eight strains of subtype H5N1 HPAIV in chickens (19), suggests that the airborne transmission through infected aerosols could require a very low dose of AIV with domestic ducks for such strains.

Infectious particles with aerodynamic diameter smaller than 10 µm are more susceptible to cause infection as they are inhaled into the lower respiratory tract. In future studies, the infectious particle size distribution should be investigated to confirm the infective potential of the exhausted air from H5N8 HPAI infected poultry facilities in case of new outbreaks, as was performed around H5N2 clade 2.3.4.4 HPAI infected poultry facilities with results indicating that viral RNA can be associated with fine particles (8, 9).

Despite the limitations of the study, our results suggest that exhaust air from H5N8 HPAI infected poultry facilities could be an important source of environmental contamination by deposition of infected dust on surfaces surrounding the infected premises, generating fomites. This phenomenon would be highly influenced by the environmental conditions such as temperature, relative humidity, UV exposure, etc. The quantity of viruses emitted in the air by an infected flock considering the downwind estimated air viral concentration and the duration of the flock excreting period (estimated, for example, at 7 days at least for the flock B) could be considered as potentially important enough to infect a nearby large poultry flock close. However, this possibility doesn’t only depend on environmental conditions but also on factors influencing infected aerosol dispersion such as wind and factors influencing animal receptivity such as species.

Our results also question the management of infected flocks. The confinement inside housing does not seem to be effective enough to prevent viral diffusion into the environment surrounding infected premises and the culling process requiring the loading of the animals into containers located outside the poultry house seems to generate an important emission of potentially infectious dust and/or aerosols into the environment. It would be essential to reduce this diffusion by rapidly implementing the depopulation using a method that reduces the air viral emission. To achieve this goal, new case management methods must require less human resource in terms of time and volume because human resources availability is the main cause of increasing time between the confirmation date and the depopulation. Furthermore, the methods must include a depopulation process minimizing the air viral diffusion to the surrounding environment. Methods such as emergency mass culling of poultry using a foam blanket over birds and in-house carcasses and litter composting could contribute to improve the control of influenza outbreaks (25, 26).

In conclusion, our results sustain the hypothesis of a potential airborne transmission contribution to the spread of the H5N8 HPAIV. However, more investigations would be required to support this hypothesis so as to provide evidence of virus viability in fine particles emitted from poultry outbreaks and epidemiological evidence.

## Author Contributions

Survey design and field implementation: AXS, RT and SLB. Laboratory analyses: PD. Data analysis: AXS, SLB, EN, AUS. Manuscript writing: AXS. Manuscript editing: SLB, EN, PD, AUS.

## Conflict of Interest Statement

The authors declare that the research was conducted in the absence of any commercial or financial relationships that could be construed as a potential conflict of interest.
